# The Ability of Flux Balance Analysis to Predict Evolution of Central Metabolism Scales with the Initial Distance to the Optimum

**DOI:** 10.1371/journal.pcbi.1003091

**Published:** 2013-06-20

**Authors:** William R. Harcombe, Nigel F. Delaney, Nicholas Leiby, Niels Klitgord, Christopher J. Marx

**Affiliations:** 1 Department of Organismic and Evolutionary Biology, Harvard University, Cambridge, Massachusetts, United States of America; 2 Systems Biology Program, Harvard University, Cambridge, Massachusetts, United States of America; 3 Bioinformatics Graduate Program, Boston University, Boston, Massachusetts, United States of America; 4 Faculty of Arts and Sciences Center for Systems Biology, Harvard University, Cambridge, Massachusetts, United States of America; ETH Zurich, Switzerland

## Abstract

The most powerful genome-scale framework to model metabolism, flux balance analysis (FBA), is an evolutionary optimality model. It hypothesizes selection upon a proposed optimality criterion in order to predict the set of internal fluxes that would maximize fitness. Here we present a direct test of the optimality assumption underlying FBA by comparing the central metabolic fluxes predicted by multiple criteria to changes measurable by a ^13^C-labeling method for experimentally-evolved strains. We considered datasets for three *Escherichia coli* evolution experiments that varied in their length, consistency of environment, and initial optimality. For ten populations that were evolved for 50,000 generations in glucose minimal medium, we observed modest changes in relative fluxes that led to small, but significant decreases in optimality and increased the distance to the predicted optimal flux distribution. In contrast, seven populations evolved on the poor substrate lactate for 900 generations collectively became more optimal and had flux distributions that moved toward predictions. For three pairs of central metabolic knockouts evolved on glucose for 600–800 generations, there was a balance between cases where optimality and flux patterns moved toward or away from FBA predictions. Despite this variation in predictability of changes in central metabolism, two generalities emerged. First, improved growth largely derived from evolved increases in the rate of substrate use. Second, FBA predictions bore out well for the two experiments initiated with ancestors with relatively sub-optimal yield, whereas those begun already quite optimal tended to move somewhat away from predictions. These findings suggest that the tradeoff between rate and yield is surprisingly modest. The observed positive correlation between rate and yield when adaptation initiated further from the optimum resulted in the ability of FBA to use stoichiometric constraints to predict the evolution of metabolism despite selection for rate.

## Author Summary

The most common method of modeling genome-scale metabolism, flux balance analysis, involves using known stoichiometry to define feasible metabolic states and then choosing between these states by proposing that evolution has selected a metabolic flux that optimizes fitness. But does evolution optimize metabolism, and if so, what component of metabolism equates to fitness? We directly tested the underlying assumption of stoichiometric optimality by comparing predicted flux distributions with changes in fluxes that occurred following experimental evolution. Across three experiments ranging in length from a few hundred to fifty thousand generations, we found that substrate uptake – an input to the model – always increased, but supposed optimality criteria such as yield only increased sometimes. Despite this, there was a clear trend. Highly optimal ancestors evolved slightly lower yield in the course of increasing the overall rate, whereas more sub-optimal strains were able to increase both. These results suggest that flux balance analysis is capable of predicting either the initial metabolic behavior of strains or how they will evolve, but not both.

## Introduction

Systems biology is beginning to provide insight into how interactions within complex networks give rise to the holistic behavior of biological systems, and how natural selection would shape these systems over the course of adaptation. Some mathematical models are made with the goal of translating known parameters of components of a small system into predictions of their function. This approach has been used to predict behavior ranging from the oscillation of natural or engineered genetic regulatory networks [1] to flow through small metabolic networks [2], [3]. For larger, genome-scale networks there is insufficient information to generate direct predictions in the same manner. Instead, one can ask how the system *should* behave were it to have already been selected to function optimally given tradeoffs between different selective criteria. One use of mechanistically-explicit optimality models is to consider the possible optimality of current biological phenomena, such as the optimality of the genetic code [4] or of the enzymatic properties of RuBisCO [5]. On the other hand, optimality models can also be used directly to predict phenotypic changes in a system that would occur over the course of adaptation, such as the evolution of virulence [6] or enzyme expression [7].

The most broadly applied metabolic modeling framework, Flux Balance Analysis (FBA), is a constraint-based evolutionary optimality model. It quantitatively predicts flux through a metabolic network that will maximize a given criterion thought to represent prior natural selection [8]. At the heart of FBA is a stoichiometric matrix, which is a mathematically transformed list of mass-balanced biochemical reactions that fully describes the known topology of the metabolic network of a cell (or other system). It is further assumed that the cell is in a metabolic steady-state, such that the sum of fluxes in and out of each internal metabolite are balanced. As additional constraints are considered (e.g., maximal flux values, irreversible reactions, biomass composition), this matrix can then be used to help define and constrain the space of feasible flux distributions in the cell. Within this feasible space, linear programing is subsequently used to solve for an optimality criterion -such as maximal biomass per substrate (see below)- to identify a feasible flux distribution that permits that optimum.

Evolutionary optimality models are powerful tools as they make it possible to build intuition about the forces that shape biological diversity. However, as has been pointed out most famously by Gould and Lewontin, they can also be misleading and can foster the wrong intuitions [9]. Optimality models make three assumptions: 1) selection (and not other processes) is the primary evolutionary force shaping a trait of interest, 2) we can identify the criterion upon which selection is acting, and 3) there are not underlying constraints which prevent a trait from being optimized. Optimality models are constructive for understanding the evolution of traits only to the extent that these assumptions can be evaluated.

FBA provides an excellent framework to generate testable hypotheses as to which selective criteria are appropriate for a given set of conditions [10], [11]. In environments such as batch culture, selection acts directly upon growth rate -as well as lag and survival in stationary phase- but not upon yield [12]. The most common optimality criterion for FBA is commonly referred to as maximizing growth rate [11]. Because this is performed by constraining one (or occasionally multiple) substrate uptake rate (S/time), this criterion is fully equivalent to predicting the maximum yield (i.e., BM/S) under the given, user-supplied substrate uptake rate. Since FBA cannot predict absolute rates of substrate uptake used as the key constraint, the question as to whether adaptation would optimize BM/S during batch culture critically depends upon the correlation between growth rate and yield. There are solid theoretical grounds to expect absolute limits to the maximization of both rate and yield of reactions [13], but it is often unclear how close biological systems are to these constraints.

In addition to maximization of biomass, various other cellular objectives have been suggested as alternative selective criteria. These include optimal energetic (rather than biosynthetic) efficiency whereby generation of ATP per substrate (ATP/S), or the minimization of the sum of fluxes (BM/Σ*v* or ATP/Σ*v*). The latter are based upon the rationale that enzymes are costly, and thus a general relationship between enzyme levels and reaction rates (although actually quite weak for any given enzyme, [14]) would lead to selection to minimize the total burden of enzymes needed. Finally it has been suggested that selection acts simultaneously upon multiple, competing criteria, leading cells to inhabit an optimal tradeoff surface known as a Pareto optimum [15], [16]. This approach constructs a surface on which no single criteria can be further increased without reducing another. It is then assumed that evolution pushes biological systems to exist somewhere on this surface. Data from a variety of experiments suggested that cells operate near to the Pareto optimum defined by BM/S, ATP/S, and minimization of Σ*v* [15].

**Figure 1:**
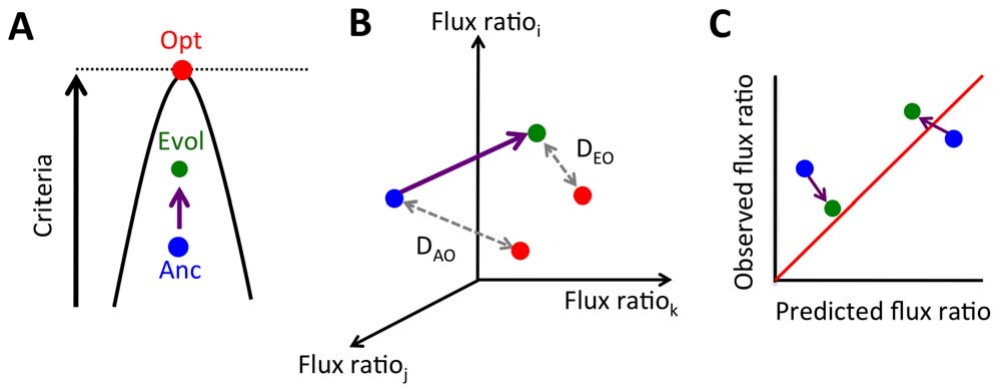
Evolution of metabolic fluxes and measures of optimality and predictability. We consider three ways to analyze changes in metabolism that relate an ancestor (Anc, blue) to an evolved isolate (E_i_, green) in regard to an FBA-predicted optimum (Opt, red). A) Evolution of metabolic fluxes can be evaluated from the perspective of changes in proximity to the theoretical maximum for a given optimality criterion (Δ% Optimality). B) A vector of flux ratios defines a position in multi-dimensional flux space. One can then consider the relative Euclidian distance of a given evolved population in this space from its optimum (D_EO_) compared to that of an ancestor from its optimum (D_AO_; plotted as log(D_EO_/D_AO_)). C) At the most detailed level, one can compare the FBA-predicted value for a given flux ratio versus that observed via ^13^C labeling.

Tests of the predictive capacity of FBA have differed in two ways depending upon: 1) whether there was *known or assumed adaptation* to the substrate in question, and 2) whether tests were a *direct or indirect comparison* of predicted internal fluxes to measured fluxes (Table 1). The majority of these tests have been conducted with *Escherichia coli*, and have assumed past selection on BM/S. The direct tests of FBA compared predicted to observed flux distributions (Figure 1) by taking advantage of empirical data generated by ^13^C-labeling techniques [17]. Briefly, this method to assay relative metabolic fluxes takes advantage of the fact that the carbon atoms of the growth substrate are shuffled in different ways by alternative metabolic pathways, and that these rearrangements leave a signature in biomass. Using gas chromatography-mass spectrometry (GC-MS) to determine the ^13^C-labeling of protein-derived amino acids, it becomes possible to infer the flux splits in the metabolic pathways leading to their synthesis [17]–[23]. Notable amongst these tests was a quantitative assessment of the relative merits of a series of optimality criteria (and constraints) in their ability to predict the intracellular fluxes of *E. coli* measured in six environments [11]. Data for wild-type cultures indicated that ATP/Σ*v* ^2^, BM/S or ATP/S were more predictive depending upon the growth condition; however, in all cases there was still significant variation between predicted and measured fluxes.

**Table 1:** Major approaches to test of FBA predictions depending upon whether there was known selection under experimental conditions and whether there was direct measurement of internal fluxes.

Past adaptation	Test of internal fluxes	Major approaches	Example papers
**Assumed**	**Indirect**	Growth rate and excretion.	Varma & Palsson, 1994 [8]
		Growth phenotypes or gene essentiality of knockouts.	[41] Raghunathan *et al*, 2009 [41]
	**Direct**	Comparison of wild-type or knockout flux pattern to mutants in one or more environments, usually using just BM/S or ATP/S as an optimality criterion.	Emmerling *et al*, 2002 [42]
		Explicit comparison of *E. coli* fluxes across environments to predictions from multiple optimality criteria.	Schuetz *et al*, 2007 [11]
**Known**	**Indirect**	Uptake, excretion, and/or growth rates for evolved strains.	Ibarra *et al*, 2002 [24]; Teusink *et al*, 2009 [43]
		mRNA and protein levels correlated with predicted pathways in FBA.	Fong *et al*, 2005 [44]; Lewis *et al*, 2010 [25]
	**Direct**	Flux changed during adaptation of *E. coli* evolved with key metabolic knockouts or on the poor substrate lactate, but no comparison made to FBA.	Fong *et al*, 2006 [20]; Hua *et al*, 2007 [28]
		Flux changes during adaptation of *E. coli* to a fluctuating environment compared to predictions of a Pareto surface.	Schuetz *et al*, 2012 [15]
		Flux measurements following 50,000 generations of *E. coli* adaptation and comparison of this and other datasets to FBA.	This study

A key advance in the use and testing of FBA came from the realization that the best test of an optimality model is to examine whether there is movement toward predicted optimal phenotypes following adaptation under known experimental conditions (Table 1). In a classic paper, populations of *E. coli* were adapted to various carbon substrates for 100–700 generations [24]. The authors ran FBA for all pairwise constraints of substrate and oxygen uptake to predict the maximal BM/S within those constraints, and what metabolites might be excreted. Remarkably, adaptation on five out of six substrates conformed to the predictions, remaining on or evolving toward a ‘line of optimality’ representing the optimal oxygen to substrate ratio. For only one of these substrates did the population move away from the predicted optimality. A follow-up study further showed that the genes expressed in evolved lines correspond to the fluxes predicted to be active by FBA [25]. Since flux changes are only sometimes well-correlated with gene expression [26], however, it remains unclear whether FBA can predict the change in internal fluxes. Although indirect, these studies have suggested that FBA might reasonably capture the evolutionary forces acting on cellular physiology and hence would be capable of predicting the outcome of evolution [27].

To our knowledge there have been only two studies in which the internal fluxes have been measured for both ancestral and evolved strains grown in a constant environment with a single growth substrate. Both involved rapid, short-term adaptation (<1,000 generations) of *E. coli* under conditions where the cultures were kept in continual exponential growth in batch culture by using frequent, large dilutions. Hua *et al* [28] measured fluxes following adaptation to the poorly-utilized substrate lactate, while Fong *et al* [20] measured fluxes following adaptation of a series of *E. coli* strains with knockouts (KOs) deleting individual enzymes of major branches of central carbon metabolism (e.g., glycolysis). Interestingly, both studies found rather divergent changes in flux distribution across replicates, and found that most improvement in growth rate was the result of increases in substrate uptake. These studies were not compared to FBA predictions, however, thus it remains unclear whether the assumed optimality criteria improved, or whether observed intracellular fluxes moved toward those predicted with a genome-scale FBA model.

In terms of using experimental evolution to test optimality, the cultures that have had the greatest time to adapt are those from the *E. coli* long-term experimental evolution (LTEE) populations that have been evolving in the Lenski laboratory for over 50,000 generations [29], [30]. These twelve replicate populations have evolved in minimal medium with glucose since 1988, experiencing 100-fold daily dilutions that result in a short lag phase, nearly seven consecutive generations in exponential phase, and then stationary phase. The LTEE experiment has enabled an unprecedented examination of genotypic and phenotypic change over an extended period of adaptation [29], [31]. Despite starting with a wild-type strain capable of rapid growth on glucose, all populations have increased dramatically in both growth rate and competitive fitness through adaptation in batch culture [32], [33]. It should be noted however, that batch culture inherently incorporates some non-steady state conditions and that improvements in lag or survival may have had pleiotropic consequences for growth. Despite this, here we ask how well FBA predictions align with the evolved changes in these populations. If FBA is unable to predict adaptation to single-nutrient, seasonal batch culture conditions we will not be able to apply it to most laboratory environments, not to mention the variable habitats experienced in nature.

The goal of the current work was to test whether the central metabolic fluxes of replicate populations of *E. coli* with known selective history in the laboratory evolved in a manner that is predictable by FBA (Figure 1). We compared the fluxes inferred from ^13^C labeling to the ranges predicted to permit optimal performance and summarize these changes in three ways: the % *optimality* possible given the inferred fluxes, the minimal *distance* in flux space between the inferred fluxes and the optimal space of distributions, and a *flux-by-flux* comparison to see how each flux changed relative to predictions. Testing the ability of optimality criteria to predict adaptation not only provides insight into the mechanisms of evolution, but also represents a critical test of the central optimality assumption of FBA. The LTEE lines began with an ancestor operating at near-optimal BM/S, but the independent populations evolved to use central metabolism less optimally. This was reflected in both a small, but statistically significant, decrease in the % optimal BM/S, and a corresponding increase in the distance from the observed to optimal flux state. In contrast, the seven lactate-evolved populations evolved to increase BM/S and moved closer to an optimal flux distribution. The three pairs of KOs had mixed results in terms of optimality and flux pattern. Overall these results indicate that evolved increases in growth rate largely resulted from increased substrate uptake. Furthermore, ancestral strains operating far from optimal yield evolved as suggested by FBA, whereas those close to the optimum experienced a modest decrease in optimality and evolved to be further from FBA predicted fluxes than their ancestor.

## Results

### Growth rate, cell dry weight and carbon uptake all increased after 50,000 generations of adaptation on glucose minimal medium

Prior to measuring internal metabolic fluxes, we first examined key growth parameters for one isolate from the 50,000 generation time-point for each of 10 independent LTEE populations (Table S1). Growth rate increased by 45% on average (Table S1), which is concordant with the 16% increase observed in these lines after 2,000 generations [32], and the 20% increase measured after 20,000 generations [33]. All evolved lines also increased their glucose uptake rates (individually significant for 5 of 10 lines: A+3, A−2, A−4, A−5, A−6; T-test, p<0.05, two-sample, equal variance throughout unless noted otherwise, Table S1), with an average increase of 18%. The cell dry weight per gram of glucose also increased by an average of 20% while max OD_600_ increased by 68%. This did not come from decreasing their excretion of organic acids, however, as acetate production actually increased by an average of 50%. No other excreted ions were observed above our limit of detection of ∼50 µM (Table S1).

### LTEE isolates have modest, but significant changes to their relative central metabolic flux distribution

**Figure 2:**
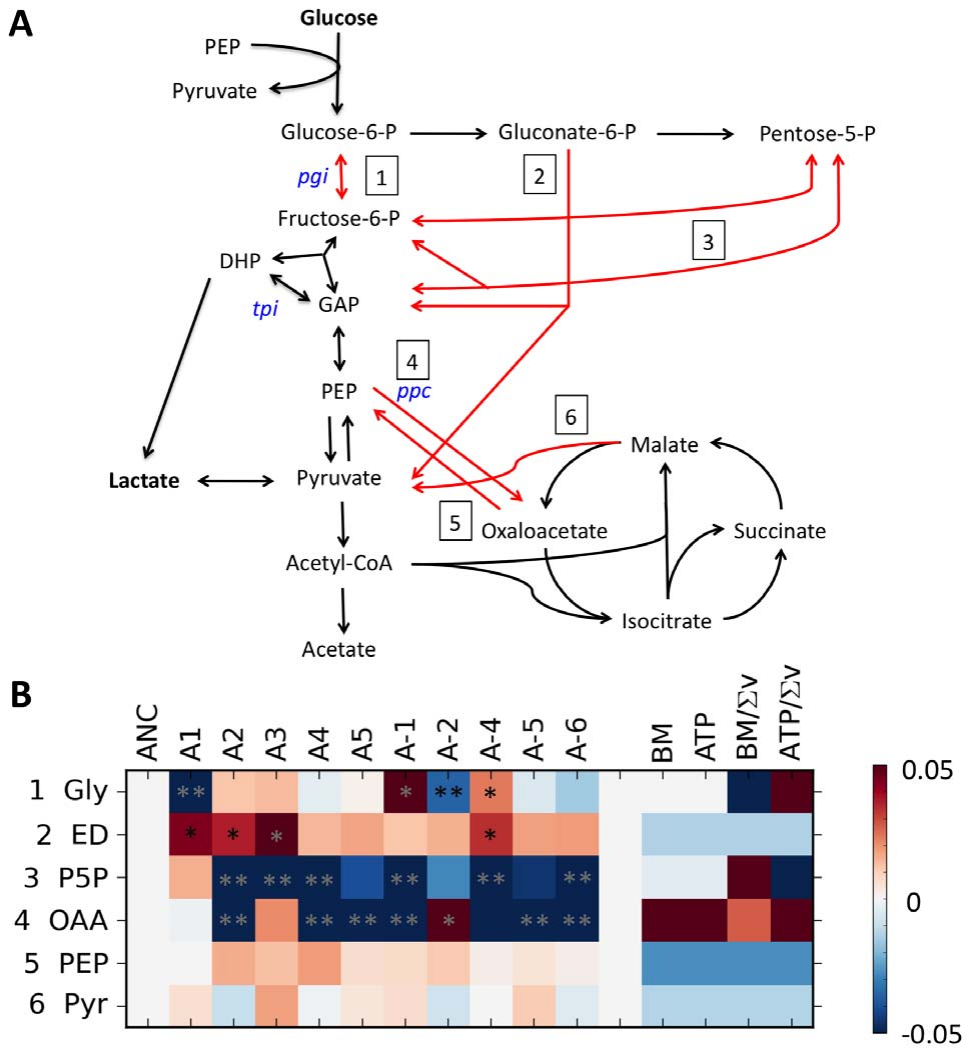
Evolved changes in central carbon metabolism for the LTEE populations after 50,000 generations of adaptation on glucose. A) The flux pathways measured for the LTEE lines are denoted with numbers and red arrows. The genes knocked out in the knockout data set and the entry point of lactate into the network are both indicated. B) A heat map of the difference between evolved and ancestral flux ratios from the LTEE populations. The right side indicates flux ratios predicted for the ancestral line according to each optimality criterion. The number of the flux ratio corresponds to the numbered pathways in A. Single asterisks denote significant changes as calculated by ANOVA, double asterisks are also significant by Tukey-HD.

In order to determine whether the improved performance of the LTEE isolates was reflected in changes in the relative use of central metabolic pathways, we used ^13^C-labeling of protein-derived amino acids [17] to infer several key flux ratios in central carbon metabolism (Figure 2A). Often the goal is to extrapolate from the measured flux ratios to calculate the flux for each reaction in a network [15], [23]. For this study, however, we limit our discussion and analyses to the flux ratios themselves, as these represent the actual number of inferences from the ^13^C-labeling data and thus each cellular branch-point is given equal weight (Text S1). It should be noted that ^13^C data for the LTEE isolates were analyzed with a program, FiatFlux [17], which is based on a simplified model of central carbon metabolism. This program was used for the previous study comparing alternate optimality criteria mentioned above [11], as well as for obtaining the flux data about the lactate [28] and KO [20] lines we analyze below. Inferences with this commonly used program are less variable than inferences based on larger models [34].

We uncovered statistically significant, but modest variation in the flux ratios of evolved isolates relative to their ancestor (Figure 2B, Table S2). In terms of the overall pattern, a MANOVA test found that flux ratios changed significantly as a function of population (Pillai's Trace = 3.80, p<0.001, Figure S1). Additionally, ANOVA tests on the flux ratios for individual lines found at least one significantly different isolate (p<0.05) for all ratios except two, and all lines had significant change in at least one flux ratio. A joint linear regression of the populations found 22 fluxes that differed from the ancestor at a p≤0.05. The False Discovery Rate (FDR) metric suggests that 18 more significant changes were found than expected by chance, whereas the more conservative Tukey HSD test finds that 10 flux changes remain significant.

A few patterns emerged in terms of the actual fluxes found to have changed in evolved isolates. First, the most parallel change was that a small, but significant portion of glucose was routed through the Entner-Doudoroff pathway (Figure 2, flux 2). In all but one case this was accompanied by a similar decrease in the proportion of carbon flowing through the pentose-phosphate pathway (flux 3). On the other hand, replicate lines evolved in opposite directions for flux through glycolysis (flux 1), and for the fluxes producing oxaloacetate from phospho*enol*pyruvate (fluxes 4). Additionally, in all cases there was no significant change in the lower bound of production of pyruvate from malate via malic enzyme (flux 6) across evolved isolates.

### Long-term evolution on glucose did not increase any optimality criterion

As a first step in testing the validity of different optimality criteria, we asked whether the flux ratios observed in evolved isolates led to increased or decreased performance with regard to each criterion (Figure 1A). The ‘*% optimality*’ can be calculated by comparing the maximum value of a criterion when the model was constrained with the observed flux ratios and substrate uptake rate to the maximum value of the criterion in the absence of the flux ratio constraints. Note that because this metric simply compares values of given optimality criteria rather than a particular set of flux ratios it is not affected by the existence of alternate optima for some fluxes.

**Figure 3:**
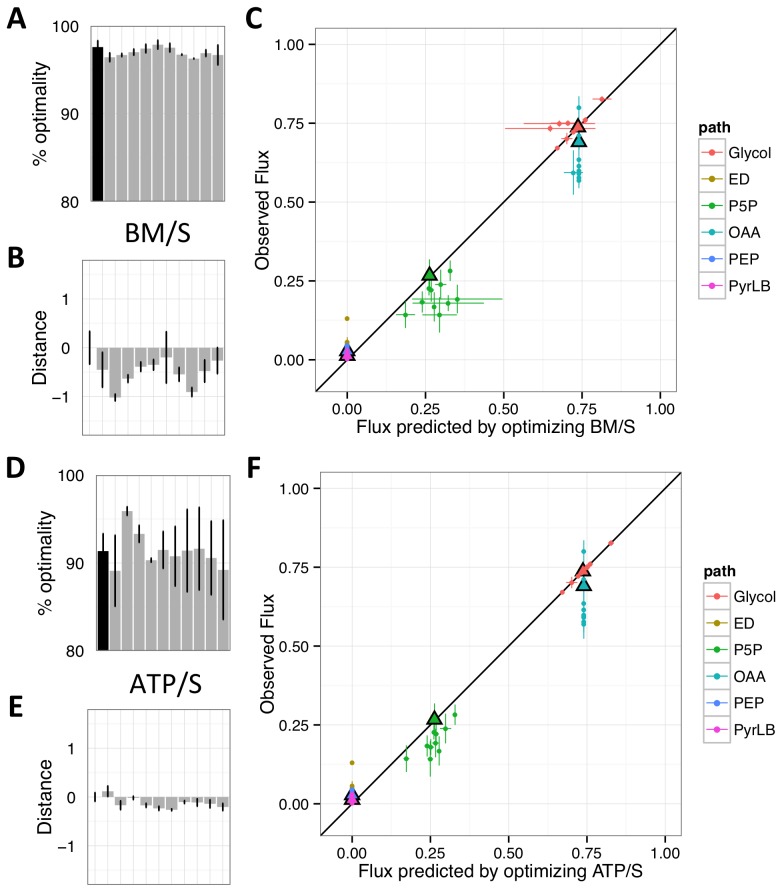
Measures of optimality and predictability after adaptation of LTEE populations to glucose for 50,000 generations. A,D) The % optimality of the ancestor (black) and evolved isolates (grey, same order as Fig. 2); B,E) distance to optimal flux distribution (plotted as log(D_EO_/D_AO_)); and C, F) comparison of predicted to observed flux ratios for FBA-predictions based upon BM/S (A–C) or ATP/S (D–F). Error bars represent standard errors of three biological replicates.

There was a slight (0.8%) but significant drop in the average percent optimal biomass production (BM/S; T-test, p = 0.008), with 9 of the 10 evolved lines decreasing relative to the ancestor (Figure 3A). Turning to alternative optimality criteria, we first found that ATP/S did not change significantly (Figure 3D), though unlike all other measures throughout, the output was not normally distributed (Shapiro-Wilk test of residuals, p = 0.002; for rest see Figures S2 and S3). Correspondingly, significance for changes in this criterion was tested with the non-parametric Mann-Whitney-Wilcoxon Rank Sum Test (p = 0.79). BM/Σ*v* and ATP/Σ*v* behaved qualitatively similarly to BM/S and ATP/S, respectively, but as neither change was significant these results are displayed only in supplementary material (Figure S4). Finally, we calculated the nearest possible flux distribution for each evolved isolate to the Pareto optimum, and found that 9 of 10 isolates were further from an optimal tradeoff between criteria than the ancestor (Figure S5).

In order to test the sensitivity of these findings to assumptions made in using FBA, we compared the effect of changing the values used for O_2_ limitation, maintenance energy, and the possible change in biomass composition that would result from the documented increase in average cell size [12]. None of these modulations changed the qualitative results and generally the default values outperformed the others (Figures S6 and S7). Therefore, the conclusion that adaptation did not lead to an increase in any optimality criterion for the LTEE populations seems rather robust.

### Long-term glucose evolution resulted in movement of the flux distribution away from predicted states

We next examined whether the flux distributions we inferred for the LTEE isolates moved toward (or away) from the flux distribution predicted to result from optimizing each criterion. We calculated the *distance* to the optimal fluxes for each evolved isolate relative to the distance between the ancestor and optimality (Figure 1B). Because the per-substrate criteria (e.g., BM/S, ATP/S) had many equally-optimal flux distributions, we identified the optimal solution that minimized the Euclidean distance from observed flux ratios. Choosing the FBA solution that is the closest to our empirical flux observations should, if anything, bias in favor of FBA.

Beginning with the overall pattern of fluxes, we quantified the log ratio of evolved to ancestral flux distance to their nearest optimum (Figure 1B). Both BM/S and ATP/S predicted optima in the opposite direction of the evolutionary flux movement, and hence evolved lines ended up significantly farther from optima than the ancestor (Figures 3B,E; S2; BM/S, T-test p = 0.0008; ATP/S, T-test, p = 0.0004). In both cases the movement away from the optimum was primarily driven by changes in the flux of oxaloaceate from phospho*enol*pyruvate.

Turning to individual flux ratios, no criterion fared particularly well (Figure 2B, 3C,F). None correctly predicted the observed increased flux through the Entner-Doudoroff pathway, nor the trend of reduced oxaloacetate from phospho*enol*pyruvate in evolved lines.

### Metabolic changes in *E. coli* evolved on the poor substrate lactate were well-predicted by FBA using BM/S as an optimality criterion

A second data set we considered was the seven populations of *E. coli* that evolved on the poorly-utilized substrate lactate for ∼900 generations [28]. These populations improved in growth rate and cell dry weight substantially (112% and 50%, respectively) in addition to increasing lactate uptake by 40% [28].

**Figure 4:**
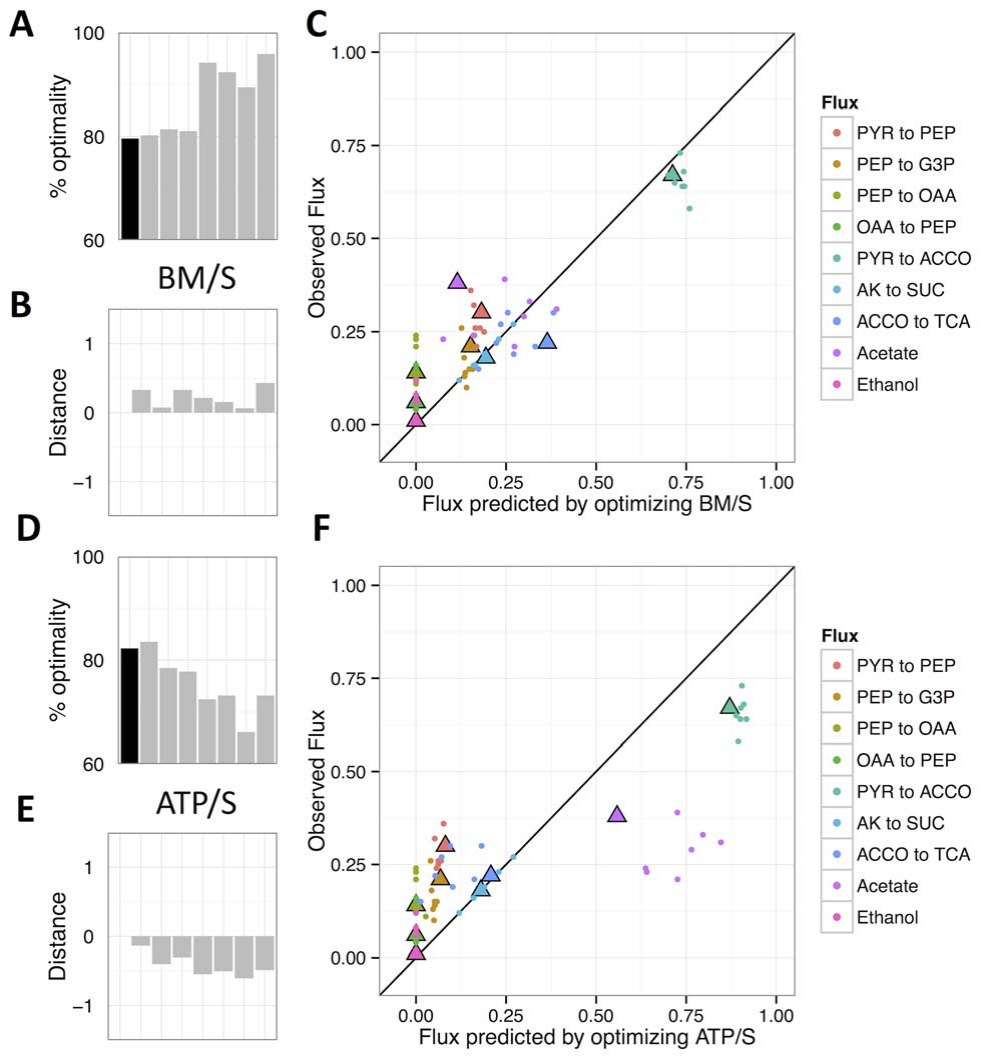
Measures of optimality and predictability after adaptation to lactate for ∼900 generations. A,D) The % optimality of the ancestor (black) and evolved isolates (grey); B,E) distance to optimal flux distribution (plotted as log(D_EO_/D_AO_)); and C, F) comparison of predicted to observed flux ratios for FBA-predictions based upon BM/S (A–C) or ATP/S (D–F).

We found that adaptation to growth on lactate led to a significant increase of 8% in the predicted percent optimal BM/S (Figure 4A; T-test, p = 0.02), whereas the % optimal ATP/S decreased significantly (Figure 4D; T-test, p = 0.01) by 7%. The % optimality for BM/Σ*v* and ATP/Σ*v* again qualitatively followed the respective per substrate criteria (Figure S4). Similarly, fluxes moved closer to the state predicted by BM/S by an average of 20% (Figure 4B; T-test, p = 0.005), largely as the result of changes in the predicted and observed flux to acetate (Figure 4C,F). In contrast, they moved away from the state predicted by ATP/S (Figure 4E; T-test, p = 0.0004). Additionally, 6 of the 7 lactate populations evolved to be further from the Pareto optimal surface than their ancestor (Figure S5).

### *E. coli* central metabolic knockouts did not evolve in the direction of FBA predictions

As a third test of whether strains evolve in a manner consistent with FBA predictions, we considered the results from evolution on glucose for KO populations with lesions in central metabolism (see Figure 2A). These data come from two populations each initiated with strains lacking phosphoglucose isomerase (Δ*pgi*), triose-phosphate isomerase (Δ*tpi*) or phospho*enol*pyruvate carboxylase (Δ*ppc*) and evolved for ∼800, ∼600, and ∼750 generations respectively [20]. Considering the improvement of these populations jointly, they increased in both growth rate and glucose uptake (172% and 157%), had large changes in central metabolic fluxes, but were largely unchanged in dry cell weight (3%). For analyzing changes in their metabolic fluxes, however, we do not present statistical tests of significance given that we only have two observations for each of these three ancestors.

**Figure 5:**
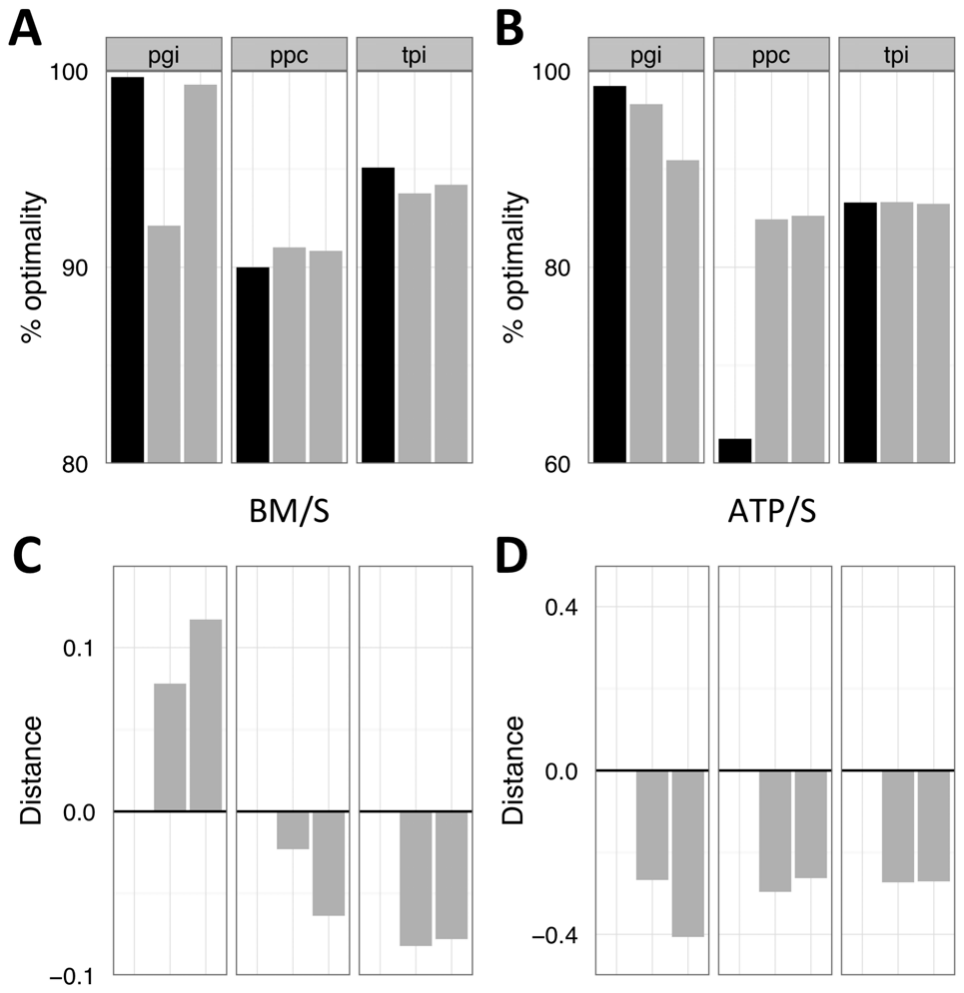
Measures of optimality and predictability after adaptation of gene knockouts on glucose for ∼600–800 generations. A,B) The % optimality of the ancestor (black) and evolved isolates (grey); C,D) distance to optimal flux distribution for FBA-predictions based upon BM/S (A,C) or ATP/S (B,D).

Our analysis of the flux data indicated that, for BM/S, Δ*pgi*, and Δ*tpi* strains got worse while Δ*ppc* strains improved their % optimality (Figure 5A). This pattern largely held for ATP/S as well, though Δ*tpi* strains showed essentially no change in % optimality (Figure 5C). The KO data set is the only one in which minimizing Σ*v* led to qualitatively different behavior from the per substrate analyses. Minimizing flux led to increases in the % optimality for *Δpgi* and *Δtpi* when using BM/Σ*v* as a criterion (Figure S4).

Evolution pushed strains further away from optima in all cases except Δ*pgi* as predicted by BM/S (Figure 5B,D). Reduced distance to the optima for Δ*pgi* was driven by reduction in the flux from oxaloacetate to phosphoenolpyruvate in evolved lines. Finally, the two Δ*pgi* evolved isolates evolved to be more Pareto optimal, the Δ*tpi* isolates were essentially equivalent to their ancestor, and the Δ*ppc* isolates became less Pareto optimal (Figure S5).

## Discussion

Genome-scale metabolism is sufficiently complex that the current state of the art in predictive models uses stoichiometry and other constraints to define the space of possible flux patterns and then suggests a given state that the cell would adopt if selection had maximized a proposed optimality criterion. The application of a mechanistic evolutionary optimality model to propose a solution to an underdetermined physiological problem is elegant and has been adopted broadly. However, there is a paucity of data testing either the central assumption that intracellular fluxes are optimized by a simple criterion, or which criterion best represents the target of selection. Here we present an analysis of metabolic evolution in the Lenski LTEE populations and make the first direct comparison of observed flux evolution to genome-scale FBA predictions.

Our analysis of the evolution of metabolic fluxes during 50,000 generations of adaptation of *E. coli* on glucose revealed changes in both the absolute and relative fluxes. Concordant with faster growth rates, we observed that all lines had increases in measured glucose uptake. Beyond this, all populations altered the way in which they utilize glucose, with significant changes in flux ratios observed across the network of central carbon metabolism. The most parallel changes in flux distribution were observed in the glycolytic pathways with a universal small, but significant increase in flux through the Entner-Doudoroff pathway, which was nearly always accompanied by a decrease through the pentose phosphate pathway. This is somewhat perplexing, as the Entner-Doudoroff pathway provides less ATP than glycolysis and no important biosynthetic intermediates. The Entner-Doudoroff pathway is shorter than glycolysis, and hence potentially less enzymatically costly. Indeed, what maintains the pathway in *E. coli* remains an open question, though it has been observed to be upregulated in *E. coli* during long-term starvation [35].

The major basis of improvement during selection upon growth rate for the LTEE populations –as was observed for the lactate and KO populations– came from increasing substrate uptake. We found that the LTEE populations continued to increase their growth rate over the 30,000 generations since it was last reported [33]. Alternative measures of yield, such as cell dry weight and OD_600_, also increased despite the slight decrease in efficiency of biomass production by central metabolism. Cell dry weight depends upon both BM/S in terms of carbon, but can also change due to the relative biomass composition of elements such as nitrogen or phosphorus. OD_600_ is even more indirect, depending upon all of these factors as well as changes in optical properties such as cell size, which is known to have increased in the LTEE [32]. We only measured flux ratios in central carbon metabolism, and thus would have missed significant adaptation that happened in peripheral metabolic pathways. Alternatively, either the bulk composition of biomass itself or the maintenance energy might change. We addressed these latter two factors in additional analyses (Figure S7), but neither of these factors significantly alters results.

Data on the evolution of central metabolism for the LTEE populations, combined with prior observations of flux evolution on lactate or by a series of three KO strains provided the opportunity to test several facets of whether the direction of evolutionary change was consistent with FBA predictions.

Across experimental systems we ascertained which proposed optimality criteria are most often consistent with the observed evolution in central metabolism. On average across five different ancestors, BM/S outperformed the other criteria in terms of either increasing or going unchanged (Figure S8). The most dramatic example was seen for the lactate-evolved populations, for which BM/S increased while ATP/S decreased. The per flux criteria (BM/Σ*v* and ATP/Σ*v*) behaved qualitatively the same as the per substrate criteria in all but two of the cases (*Δpgi* and *Δtpi*). BM/Σ*v* outperformed BM/S in these two cases, but, for example, did not significantly improve in the lactate populations. The data also suggest that cultures quite often evolved to be further from their Pareto optimum representing the space of optimal tradeoffs [15], with 19 of 23 populations in total moving further from the Pareto surface than their respective ancestral genotypes. These results suggest that optimal biomass yield –which is the most commonly utilized criterion for FBA– was the best overall stoichiometric proxy for cultures where selection was directly upon growth rate. It will be quite interesting to analyze populations grown in a manner where yield (BM/S) is directly selected.

**Figure 6:**
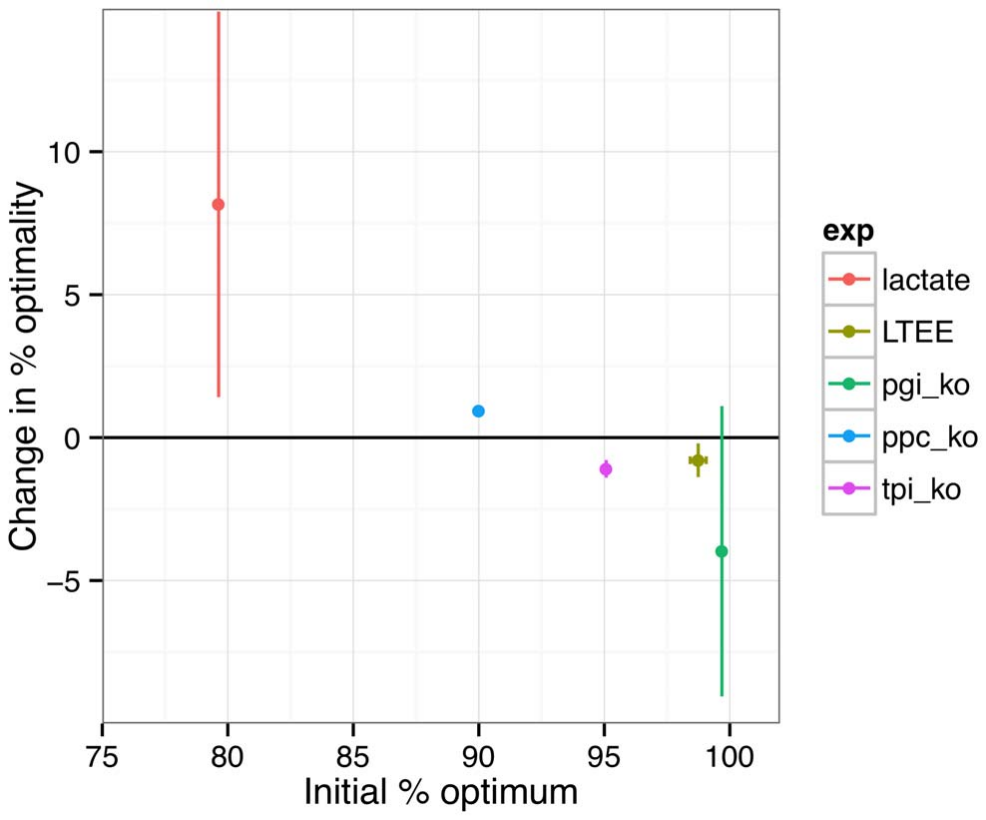
Evolutionary change in % optimality versus initial % optimality of the ancestor across data sets for BM/S. Error bars represent standard errors between evolved populations.

Overall, approximately half of the flux data were consistent with FBA predictions, and half refuted the common assumption that evolution acts to optimize efficiency; what accounts for this discrepancy? The major factor that appears to account for this difference is the initial degree of optimality for the ancestor of the evolved lines (Figure 6). For the lactate and Δ*ppc* populations, which began at approximately 80% and 90% optimality for BM/S, all 9 total replicates increased in BM/S. On the other hand, 13 of 14 populations starting at or above 95% efficiency –LTEE and the other two KOs– decreased in BM/S. A negative correlation holds whether one performs a parametric statistical test (Pearson correlation, p<0.0001) or a non-parametric Spearman correlation coefficient (p<0.0001), though it should be noted that the strength of the correlation is largely driven by the lactate data set.

The finding that selection on optimal efficiency depends on distance to the optimum is both of practical and fundamental interest. The analysis represents the first direct demonstration that FBA can be used to predict changes in intracellular metabolism that result from adaptation on a single carbon source. This positive result comes with the caveat that strains must begin far from the optimum. Systems initially operating at high yield –like the LTEE and the Δ*pgi* strains that both began above 98% optimal– may end up evolving to be further from optimal than they began. In other words, this suggests one can either predict the initial physiological state or the direction of evolution, but not both.

What is perhaps the most remarkable about these findings is that even for cultures with a negative correlation between rate and yield, the tradeoff was quite modest. Small decreases in BM/S were more than made up for by large increases in uptake, leading to a net increase in growth rate despite mild antagonism. Given that there is no direct selection upon yield during batch culture, this perhaps suggests the existence of constraints upon the further improvement of substrate uptake. As long as uptake is held constant then changes in yield would directly translate into changes in growth rate. As such, this would maintain purifying selection upon yield, even over 50,000 generations. On the other hand, the low efficiency ancestors were able to evolve both improved substrate uptake and yield simultaneously.

Although FBA is typically applied as a practical tool to guide experiments –and it has had some remarkable successes, such as correctly predicting a rather unexpected new metabolic pathway in some cancers [36]– it also serves as a quantitative, testable, falsifiable model that connects physiology to evolution. The interplay of optimality models and laboratory adaptation will be critical as the field continues to move toward a fuller understanding of the selection and constraints that act upon biochemical networks.

## Materials and Methods

### Strains and growth conditions during selection

*Escherichia coli* B isolates were obtained from the Lenski LTEE experiment [29] after 50,000 generations. Briefly, 12 populations of *E. coli* were founded with either the arabinose-negative strain REL606 (populations A−1 to A−6) or the arabinose-positive derivative, REL607 (A+1 to A+6). These were evolved in 10 mL of Davis-Mingioli minimal medium with 139 µM glucose (25 mg/L) as a growth substrate in 50 mL flasks since 1988. These lines have been cultured at 37°C while shaking at 120 rpm and have been transferred daily via 1∶100 dilutions (∼6.64 net doublings per day).

The isolates analyzed in the current experiment consisted of the ancestral line, REL606 [29], as well as the ‘A’ clone from 10 of the 12 lines frozen at 50,000 generations that were used in an earlier paper (A−1 = REL11330; A−2 = REL11333; A−4 = REL11336; A−5 = REL11339; A−6 = REL11389; A+1 = 11392; A+2 = REL11342; A+3 = REL11345; A+4 = REL11348, A+5 = REL11367) [30]. The A−2 clone used is from the ‘large’ lineage that has coexisted with a cross-feeding ‘small’ lineage for tens of thousands of generations [37]. The isolate from the citrate-consuming population A−3 (REL11364) was not used because it adapted to citrate consumption in addition to glucose [38]. The A+6 isolate (REL11370) was excluded from analysis because it had inconsistent growth, and gave irregular flux data. This population was previously excluded from a study of growth rate vs. yield at 20,000 generations for similar reasons [13].

### Measurement of key metabolic flux ratios

Flux measurements were obtained based on the methods of Zamboni *et al* [17]. Evolved isolates were grown in 150 mL of Davis-Mingioli minimal media with 139 µM glucose without sodium citrate (excluded to ensure that it was not used as a secondary carbon source by any line). In order to obtain information from different parts of central metabolism, ^13^C-labeling either utilized a 20∶80 ratio of [U-^13^C]labeled∶unlabeled glucose or 100% [1-^13^C]glucose (Cambridge Isotope Laboratories, Andover, MA). The ancestral REL606 was grown in 200 mL to obtain sufficient cell material. At mid-log phase (60–80% max OD) all cells were pelleted from the media, hydrolyzed overnight in 6 M HCl, and dried. The dry cell material was then derivatized for an hour at 85°C with 40 µL each of dimethylformamide and *N*-*tert*-butyldimethylsilyl-*N*-methyltrifluoroacetamide with 1% *tert*-butyldimethyl-chlorosilane. The derivatized cell material was injected into a Shimadzu QP2010 GCMS (Columbia, MD). The injection source was 230°C. The oven was held at 160°C for 1 min, ramped to 310°C at 20°C min^−1^, and finally held at 310°C for 0.5 min. Flow rate was 1 mL min^−1^ and split was 10. The column was a 30 m Rxi-1ms (Restek, Bellefonte, PA). Three technical and three biological replicates were run for each isolate.

Data files from the GC-MS were analyzed in FiatFlux [17], as had been used for the lactate [28] and KO [20] populations we also analyzed. The data conversion files were rewritten to load the raw spectra produced by our MS. Following the established protocol, uninformative amino acid fragments were removed. Means for each biological replicate were calculated from the average of three technical replicates. Shapiro-Wilk tests were used to validate the assumption of normally-distributed errors for estimated flux ratios for each strain (Figures S2, S3). Variance in flux ratios was then analyzed with a MANOVA test using the Pillai's Trace metric with flux ratios entered as separate dependent variables (Figure S1). Univariate ANOVA tests were also run to investigate which of the measured flux ratios changed significantly for individual strains.

The flux of oxaloacetate (OAA) from phospho*enol*pyruvate (PEP) was further estimated by a Monte-Carlo method to determine the contribution of the glyoxylate shunt. The method follows Waegeman *et al*, 2011 [23] and uses MATLAB code they kindly provided. In short, average mass distribution vectors and standard deviations were calculated from the measured samples. The ‘normrand’ function was then used to randomly draw from these mean distributions 1000 times. For each draw, a grid search was used to find the flux ratios that best fit the mass distribution vectors. Substantial variation was found for the fraction of labeled CO_2_ and flux through the glyoxylate shunt, but in all cases there was very strong support for the flux ratio of oxaloacetate from phospho*enol*pyruvate that had previously been calculated by FiatFlux.

### Physiological measurements of growth rate, cell dry weight, glucose uptake and acetate excretion

Uptake and production of cell material were determined in a separate set of experiments. In these experiments glucose concentrations were increased ten-fold to 1.39 mM so that enough of the compounds would be present to measure precisely. A volume of 250 µL of overnight culture was inoculated into 50 mL of media grown in a 250 mL flask at 225 rpm. Growth rate was determined by fitting a logarithmic model to OD_600_ measurements. A 10 mL sample was removed at early (OD_600_ of 0.090–0.120) and late (OD_600_ of 0.275–0.400) log phase. Cells were immediately removed from the media by passage through a 0.2 µM filter. Glucose concentrations were determined in the spent media using a glucose oxidase assay kit (Sigma, Saint Louis, MO). Acetate concentrations were determined by ion chromatography with a Dionex ICS-200 RFIC. The flow rate was 1.5 ml/min and the column temperature was 30°C. Cell dry weight (CDW), was measured as the mass of the pellet from 100 mL of fully-grown culture after overnight lyophilization. Three replicates were assayed for each measurement.

### Calculation of variation in flux ratios across evolved isolates

The degree of parallelism between replicates in the evolution of flux ratios was determined by calculating the coefficient of variation in flux ratios. For each flux ratio the standard deviation between evolved replicates was divided by the mean of that flux ratio. This value was then averaged across all flux ratios. Values close to zero indicate a high degree of similarity between evolved lines.

### Prediction of FBA optima

Flux analysis was carried out with a genome-scale model of *E. coli* metabolism (iAF_1260 [39]). The model incorporates 2382 reactions and 1668 metabolites. Substrate uptake and excretion were constrained to that observed, otherwise the default minimal media environment was used. The lower bound on maintenance energy was left at the default value of 8.9 mmol ATP/g/hr. Oxygen uptake rates were set to those observed for the lactate strains; however these data were not available for the REL or KO strains. In these cases, oxygen uptake for the ancestor was scaled across the previously observed range of 11.5–14.75 mmol/gCDW/hr [11]. Previous work demonstrated that oxygen uptake varies as a function of evolution, but that the ratio of substrate to oxygen usage remained largely constant [24]. Oxygen constraints for evolved lines were therefore set based on evolved glucose uptake rates and the ancestral ratio of oxygen/glucose. Changing the value of ancestral oxygen constraint, or the slope of constraint line had little qualitative effect (Figure S6), so just the results based on an ancestral uptake of 14.75 mmol/g/hr and a slope maintaining the original oxygen/glucose rates are reported in the text. Gene knockouts were simulated by constraining flux through the missing gene to zero.

For all data sets we systematically tested the predictive ability of four different optimality criteria: max biomass per unit substrate (BM/S), max ATP per unit substrate (ATP/S), max biomass per unit flux (BM/Σ*v*) and max ATP per unit flux (ATP/Σ*v*). These criteria relate to the best performers in Schuetz *et al* 2007 [11] and were defined as in that study. The per-substrate criteria maximized the criterion and then subsequently chose a flux distribution that minimized the difference from the observed isolate ratios. This process always provides a flux distribution with maximal production of ATP (or biomass). The per-flux criteria optimize the ratio of ATP (or biomass) to the sum of the flux. Optimizing this ratio leads to a single optimal flux solution that often produces less than the maximal ATP (or biomass). For ATP criteria, flux to excess ATP use (via maintenance energy) was maximized while constraining the lower limit of biomass production to the ancestral growth rate.

Minimizing the distance between observed and predicted optimal flux distributions was accomplished by minimizing a distance term. Flux ratios can be constrained by adding a row to the S matrix such that: 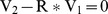
Where V_n_ is the flux through reaction n and R is the ratio V_2_/V_1_. To minimize distance between observed and predicted ratios the equation becomes:
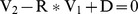


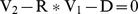
Where D represents distance from the observed ratio and is added as two columns to the S matrix (and concomitant rows in the flux vector). Biomass or ATP can be constrained to its maximum value and then the flux distribution that is closest to observed values can be calculated by running linear optimization minimizing D as the objective function.

### Comparison of experimental flux ratios to FBA-predicted optima

We first tested whether flux ratios evolve to increase each selective criterion. The optimal value of each criterion was compared against the maximum value of the criterion when the model was constrained to have the experimentally observed flux ratios. Percent optimality, calculated as the constrained criterion divided by optimal criterion, was determined for the ancestor and evolved lines.

For the LTEE lines the constrained flux ratios were serine through glycolysis, pyruvate through Entner-Doudoroff, oxaloacetate from phospho*enol*pyruvate, phospho*enol*pyruvate from oxaloacetate, and the pyruvate from malate. The ratios were calculated following Fischer and Sauer 2003 [40]; the exact equations used are provided in the supplementary material (Table S3). Each ratio was constrained by adding a row to the S matrix that defined the relationship between relevant fluxes (as described in the first equation of the previous section). The ratio inferred for pyruvate from malate was treated either as an absolute constraint or a lower bound but because all optimality criteria push this value towards 0 the results were equivalent.

To propagate uncertainty in glucose uptake, acetate excretion and flux ratios for the LTEE isolates, separate calculations of properties such as BM/S were made for each of 3 biological replicates, which themselves represented the average of 3 technical replicates. The mean and standard error for optimality metrics was calculated for each strain from the biological replicates.

Flux constraints for lactate and knockout data sets were implemented as upper and lower bounds, because reported flux ratios were relative to substrate uptake rather than other internal fluxes. Lactate adaptation lines were constrained to have flux ratios ±5% of the values reported in Hua *et al* 2007 [28]. Gene knockout lines were constrained with the flux ratios and errors reported in Fong *et al*, 2006 [20].

To determine whether strains evolved towards predicted optimal intracellular physiologies we used a standardized metric to ask if evolved lines were closer to an optimal solution than the ancestor. This distance metric was calculated as:
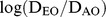
where D_EO_ was the distance of the evolved flux ratios from the closest optimal solution, and D_AO_ was the distance of the ancestor from its closest optimal solution. Distances were calculated as Euclidean distance between the flux ratios observed in each data set and those predicted. It should be noted that because optimal flux ratios change with substrate uptake the ancestral and evolved optima were different points. The metric is 0 if the evolved isolate distance has not changed relative to the ancestor, increasingly positive as the evolved strain moves nearer an optimum, and increasingly negative as it moves further away.

### Pareto optimality

A Pareto optimal surface was calculated for each line by constraining the substrate uptake rate and then doing a nested grid search [15]. A grid search across the range of feasible biomass values was executed. At each value of biomass a grid search of ATP yields was carried out and the sum of fluxes was subsequently minimized at every interval. Conservatively, for each isolate we then determined the closest possible position to its optimal surface given the observed constraints. Distance between the isolate and the Pareto optimal surface was calculated from the difference in standardized criteria.

### Statistical tests

The normality assumption for physiological measurements for the LTEE populations and optimality metrics for all data sets were checked with the Shapiro-Wilk test on the residuals of the linear model fitting the metric against strains. In all but one case the null hypothesis that the distribution was normal could not be rejected at p<0.05. The % optimality for the LTEE lines with ATP/S as the optimality criterion was not normally distributed. Q-Q plots are presented in the supplementary material (Figures S2 and S3).

For the LTEE lines ancestral versus evolved values were compared with two-sided, two sample T-tests assuming equal variance. For the non-normal ATP/S comparison a Mann-Whitney Wilcoxon Rank Sum Test was used instead. For the lactate populations only a single value was available for the ancestor so two-sided, one-sample T-tests were performed testing against the ancestral value as the mean.

## Supporting Information

Figure S1 **Covariance of fluxes inferred for the LTEE.** To determine whether there was a significant change in flux ratios between populations of the LTEE we ran a MANOVA as described in the text; however, to provide further insight into the basis of the significant differences that we observed we present a chart of the correlations between all fluxes. A) The value of the correlation and the significance are presented on the bottom half of the chart. B) The proportion of variation explained by each eigenvector.
(PDF)

Figure S2 **Normality tests for data associated with the LTEE.** Q-Q plots and Shapiro-Wilk values are displayed for growth parameters, and flux ratios. Additionally, data is displayed about the normality of % optimality and distance for different criteria.
(PDF)

Figure S3 **Normality tests for data associated with the lactate strains.** Q-Q plots and Shapiro-Wilk values are displayed for growth parameters, and flux ratios. Additionally, data is displayed about the normality of % optimality and distance for different criteria.
(PDF)

Figure S4 **Measures of optimality based upon BM/Σ** ***v*** **or ATP/Σ** ***v*** **for all data sets.** (A,B,E,F,I,J) The % optimality of the ancestor (black) and evolved isolates (grey); (C,D,G,H,K,L) distance to optimal flux distribution for FBA-predictions (plotted as log(D_EO_/D_AO_)). These were performed based upon BM/Σ*v* (A,C,E,G,I,K) or ATP/Σ*v* (B,D,F,H,J,L). The data sets are LTEE (A–D), lactate (E–H), and KO (I–L). Error bars for LTEE represent standard errors of three biological replicates.
(PDF)

Figure S5 **Measures of optimality based on maximizing the tradeoff between BM, ATP and Σ** ***v*** **for all data sets.** The Pareto distance of the ancestor (black) and evolved isolates (grey) for LTEE (A), lactate (B), and KO (C). Error bars represent standard errors of three biological replicates.
(PDF)

Figure S6 **Implementation of oxygen constraints.** Following the example of Schuetz *et al* 2007 [11] we varied the ancestral oxygen uptake rate across the range reported in the literature (11.5–14.75 mmol/g hr). Ibarra *et al* 2002 [24] report that the ratio of oxygen to glucose uptake remains largely constant as cells evolve. We tested the impact of varying ancestral oxygen/glucose ratio as well as the slope of evolutionary change from 0.5 to 1.5. There was no significant difference in the change in % optimality for either BM/S (A) or ATP/S (B) across this wide range of parameter values. Results are not presented for an ancestral oxygen uptake rate of 11.5 for ATP/S because this constraint caused infeasible solutions for several evolved populations. Results obtained with the default values used throughout the manuscript, an ancestral uptake of 14.75 mmol/g hr and a slope of 1, are highlighted in red.
(PDF)

Figure S7 **The effect that potential evolution of constraints would have on average change in % optimality between ancestor and evolved lines.** A) Lipid content was altered in evolved lines from 80–120% of the default values. B) Maintenance energy in evolved lines was altered from 50–150% of the default value of 8.39 mmol/g hr. Analyses for ATP/S are not shown, as setting a lower bound on maintenance energy has no effect if ATP production is being maximized. Results for simulations run with default (red) and altered (blue) constraints are shown for the LTEE set when optimized for either BM/S or ATP/S. Error bars represent standard errors between replicate lines.
(PDF)

Figure S8 **Average difference in % optimality between ancestor and evolved lines for each data set for each criterion.** The criteria tested were BM/S (blue), ATP/S (red), BM/Σ*v* (green) and ATP/Σ*v* (purple). Error bars represent standard deviations of replicate lines.
(PDF)

Table S1 **Growth parameters for ancestral and evolved LTEE isolates.**
(PDF)

Table S2 **Experimentally determined flux ratios for ancestral and evolved LTEE isolates.** PEP through PPP is an upper bound (ub); PYR from MAL is a lower bound (lb).
(PDF)

Text S1 **Equations used to calculate flux ratios.** The notation v(x) represents the flux through reaction x of the iaf1260 genome-scale model of metabolism.
(PDF)
