# Supplementary figures and images for: The Ability of Flux Balance Analysis to Predict Evolution of Central Metabolism Scales with the Initial Distance to the Optimum
Source: PLoS Comput Biol. 2013 Jun 20;9(6):e1003091. doi: 10.1371/journal.pcbi.1003091 (PMC3688462; doi:10.1371/journal.pcbi.1003091)

Figure S1

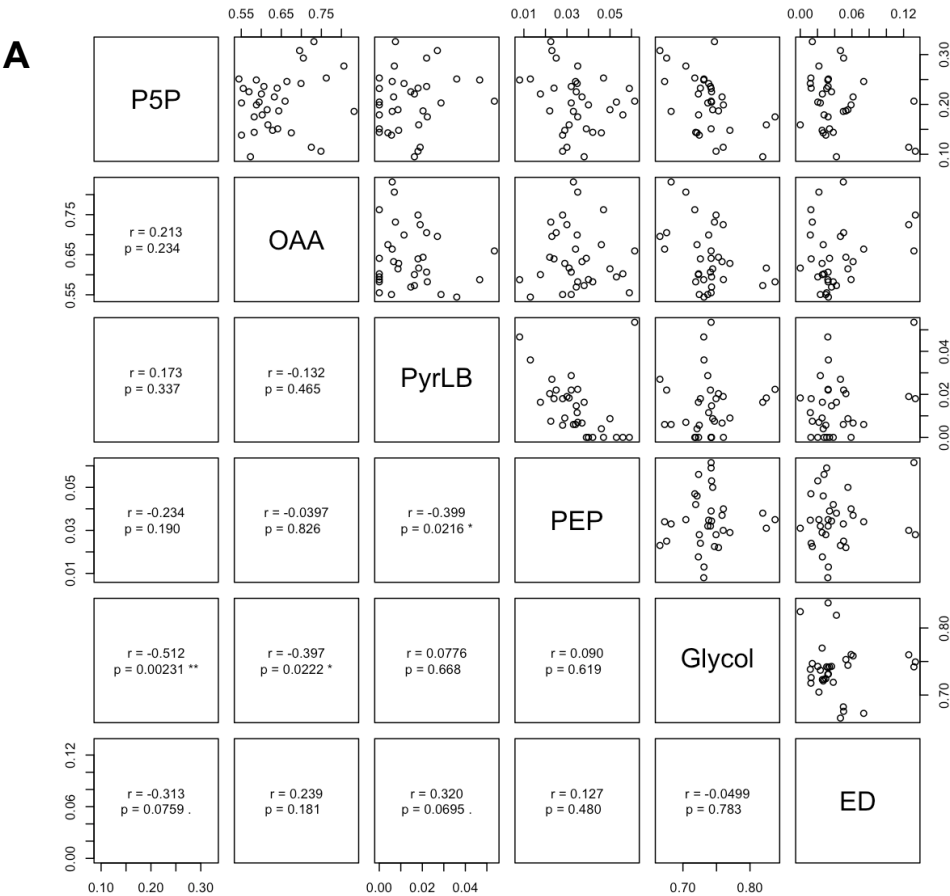

**B** Proportion of Variation explained by each eigenvector

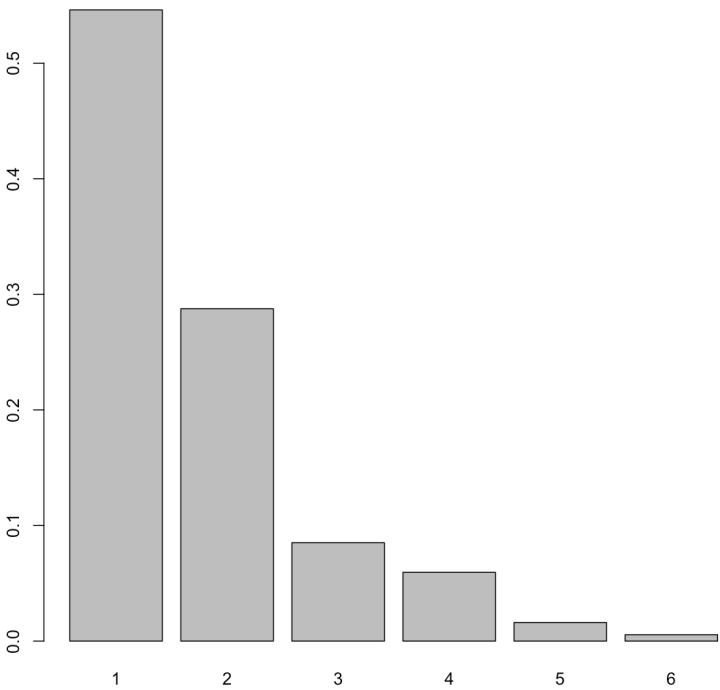

Supplement: Figure S1 — Covariance of fluxes inferred for the LTEE. To determine whether there was a significant change in flux ratios between populations of the LTEE we ran a MANOVA as described in the text; however, to provide further insight into the basis of the significant differences that we observed we present a chart of the correlations between all fluxes. A) The value of the correlation and the significance are presented on the bottom half of the chart. B) The proportion of variation explained by each eigenvector. (PDF) [file pcbi.1003091.s001.pdf]

**Figure S2**

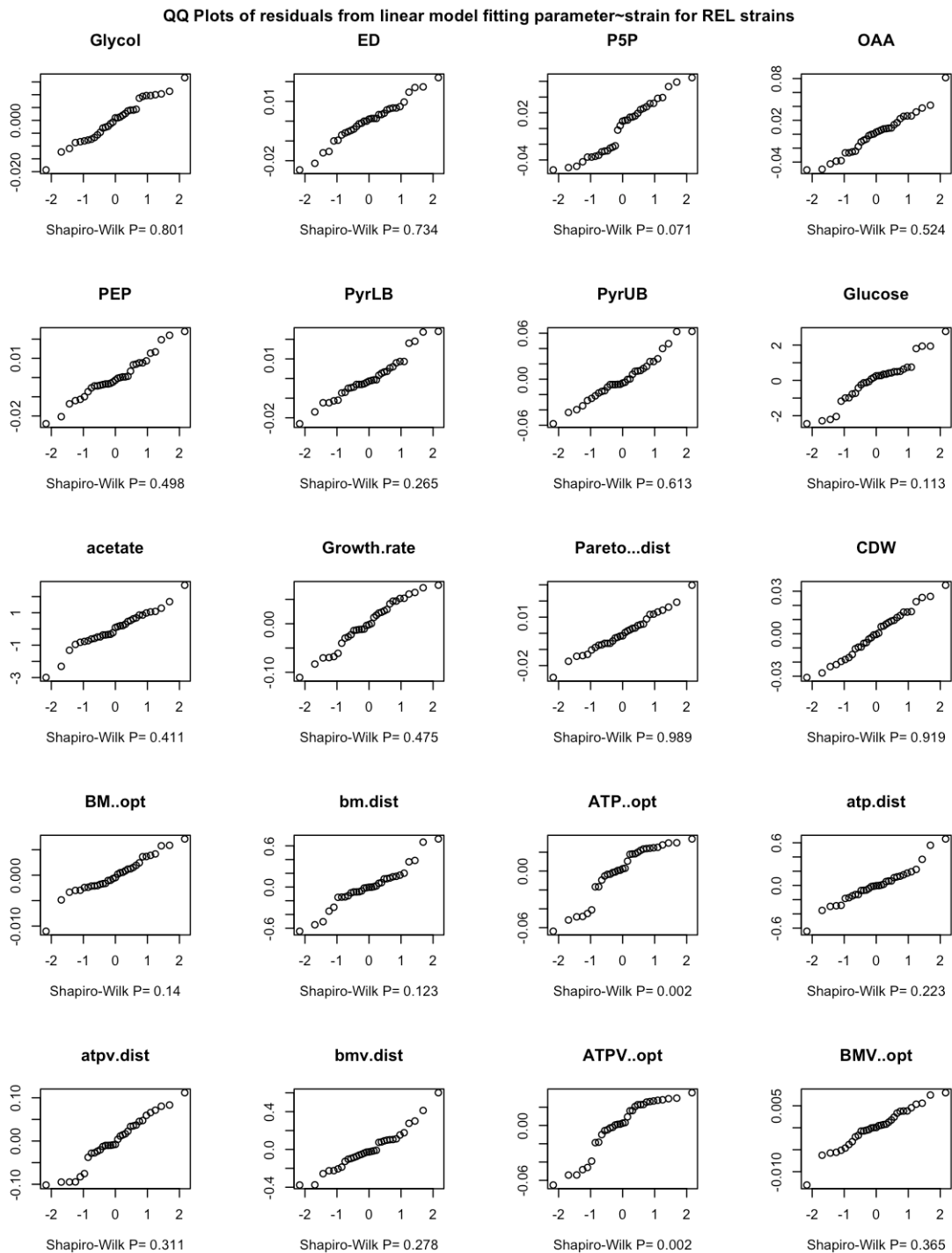

Supplement: Figure S2 — Normality tests for data associated with the LTEE. Q-Q plots and Shapiro-Wilk values are displayed for growth parameters, and flux ratios. Additionally, data is displayed about the normality of % optimality and distance for different criteria. (PDF) [file pcbi.1003091.s002.pdf]

# Figure S3

QQ Plots of residuals from linear model fitting parameter~strain for Lactate strains

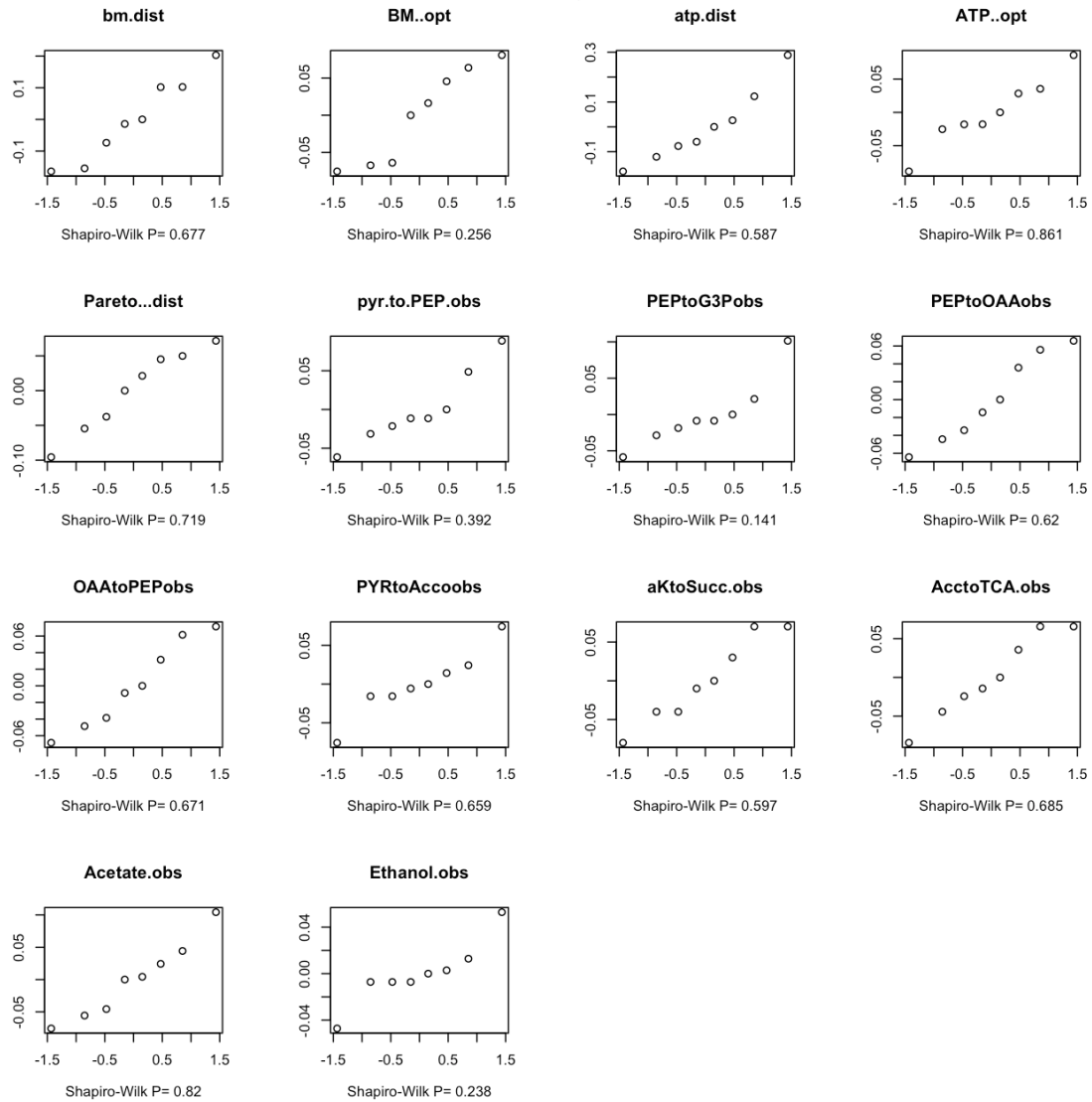

Supplement: Figure S3 — Normality tests for data associated with the lactate strains. Q-Q plots and Shapiro-Wilk values are displayed for growth parameters, and flux ratios. Additionally, data is displayed about the normality of % optimality and distance for different criteria. (PDF) [file pcbi.1003091.s003.pdf]

**Figure S4**

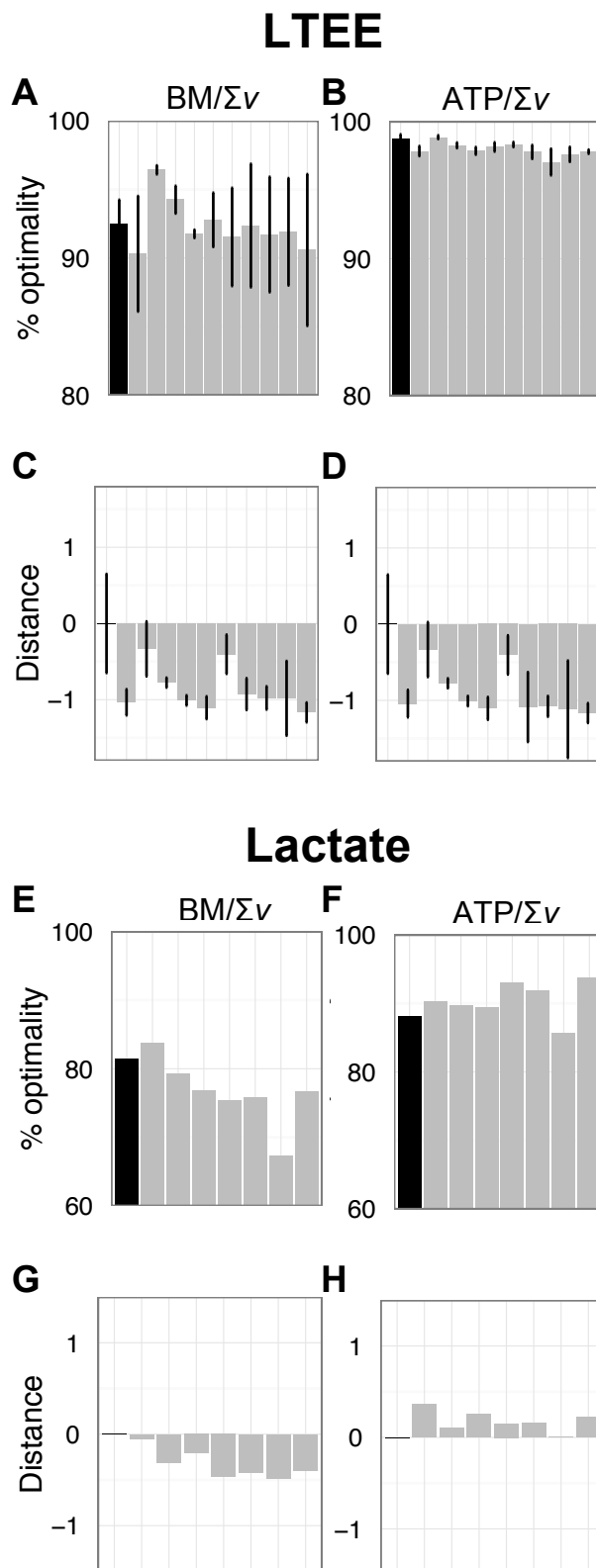

# KO

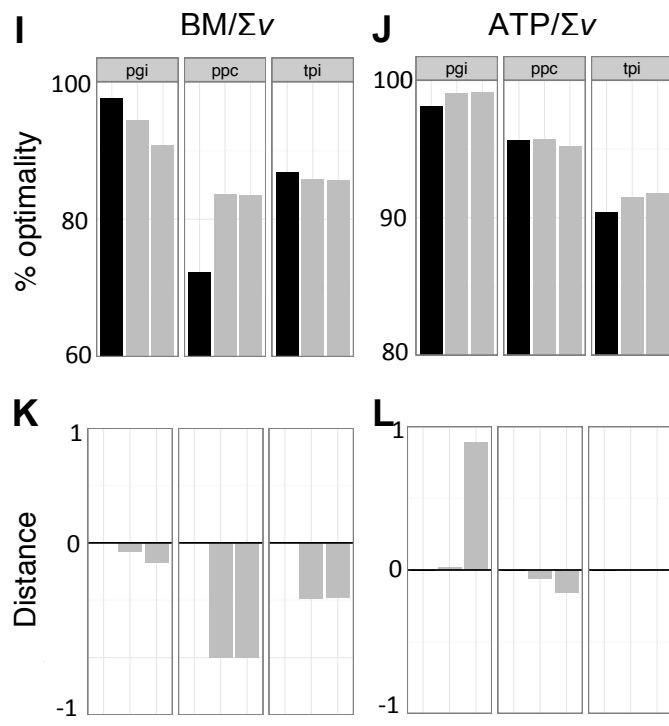

Supplement: Figure S4 — Measures of optimality based upon BM/Σ v or ATP/Σ v for all data sets. (A,B,E,F,I,J) The % optimality of the ancestor (black) and evolved isolates (grey); (C,D,G,H,K,L) distance to optimal flux distribution for FBA-predictions (plotted as log(DEO/DAO)). These were performed based upon BM/Σv (A,C,E,G,I,K) or ATP/Σv (B,D,F,H,J,L). The data sets are LTEE (A–D), lactate (E–H), and KO (I–L). Error bars for LTEE represent standard errors of three biological replicates. (PDF) [file pcbi.1003091.s004.pdf]

Figure S5

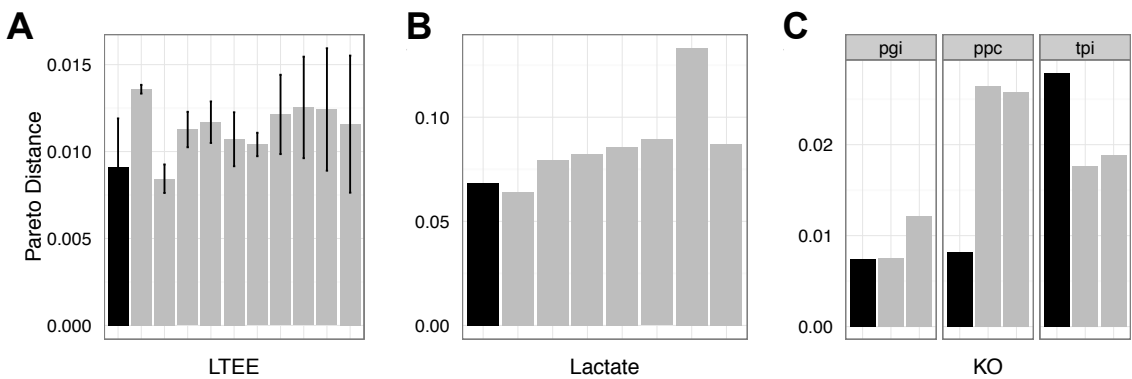

Supplement: Figure S5 — Measures of optimality based on maximizing the tradeoff between BM, ATP and Σ v for all data sets. The Pareto distance of the ancestor (black) and evolved isolates (grey) for LTEE (A), lactate (B), and KO (C). Error bars represent standard errors of three biological replicates. (PDF) [file pcbi.1003091.s005.pdf]

Figure S6

A

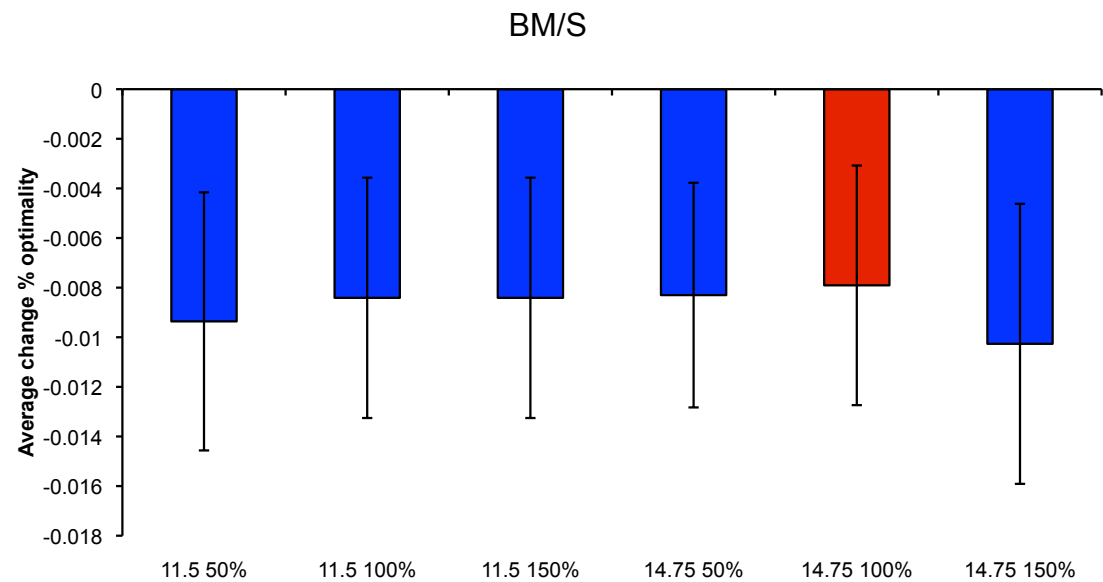

B

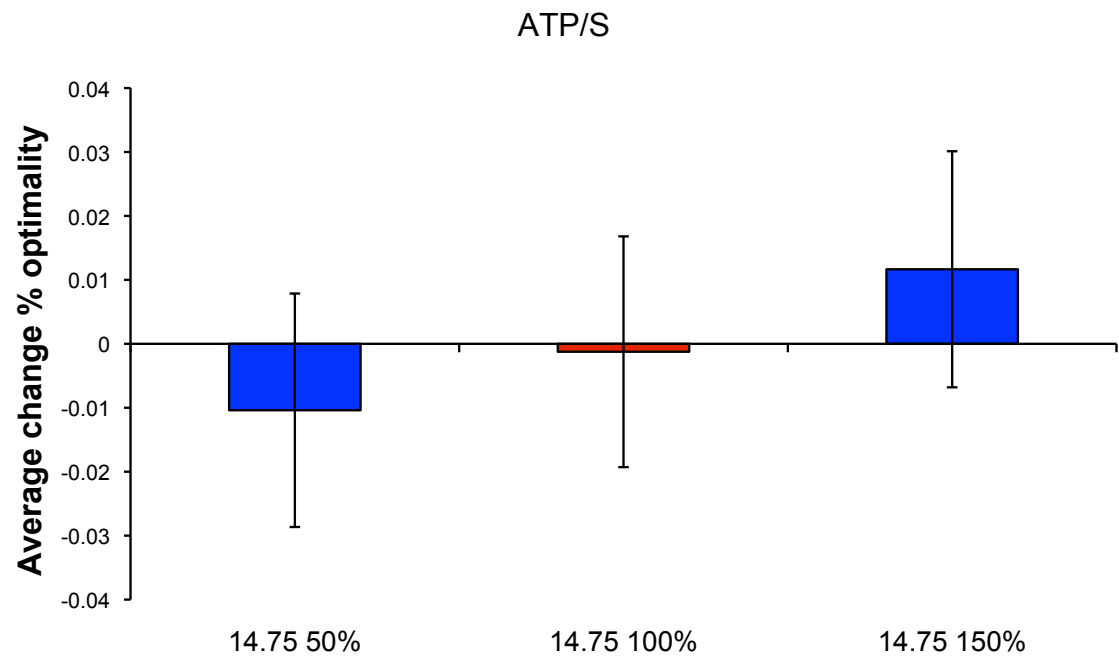

Supplement: Figure S6 — Implementation of oxygen constraints. Following the example of Schuetz et al 2007 [11] we varied the ancestral oxygen uptake rate across the range reported in the literature (11.5–14.75 mmol/g hr). Ibarra et al 2002 [24] report that the ratio of oxygen to glucose uptake remains largely constant as cells evolve. We tested the impact of varying ancestral oxygen/glucose ratio as well as the slope of evolutionary change from 0.5 to 1.5. There was no significant difference in the change in % optimality for either BM/S (A) or ATP/S (B) across this wide range of parameter values. Results are not presented for an ancestral oxygen uptake rate of 11.5 for ATP/S because this constraint caused infeasible solutions for several evolved populations. Results obtained with the default values used throughout the manuscript, an ancestral uptake of 14.75 mmol/g hr and a slope of 1, are highlighted in red. (PDF) [file pcbi.1003091.s006.pdf]

Figure S7

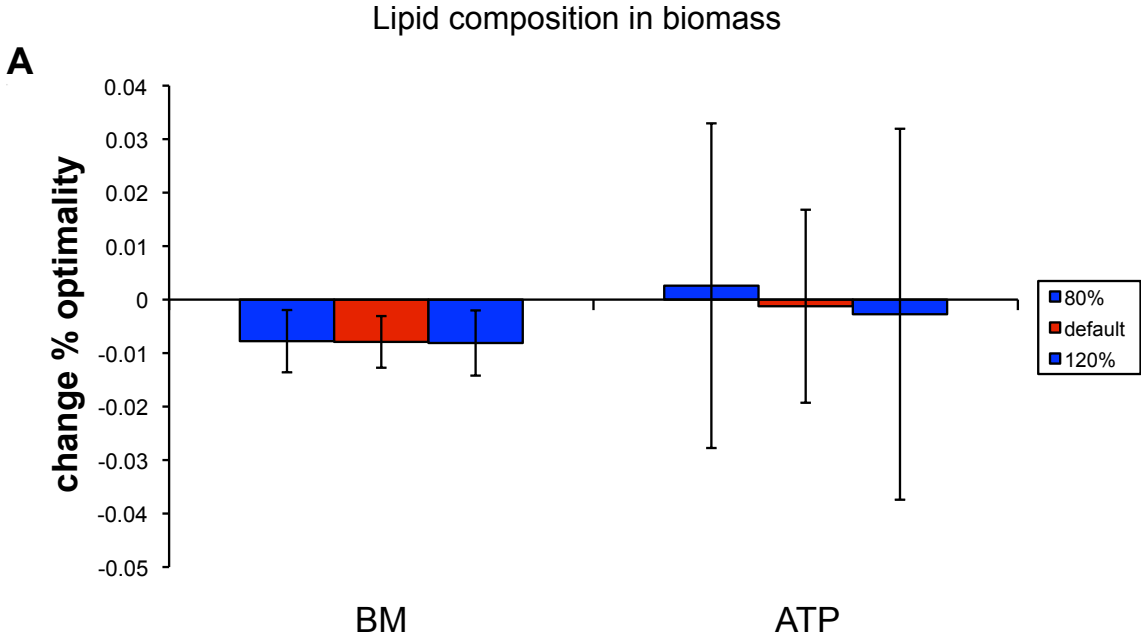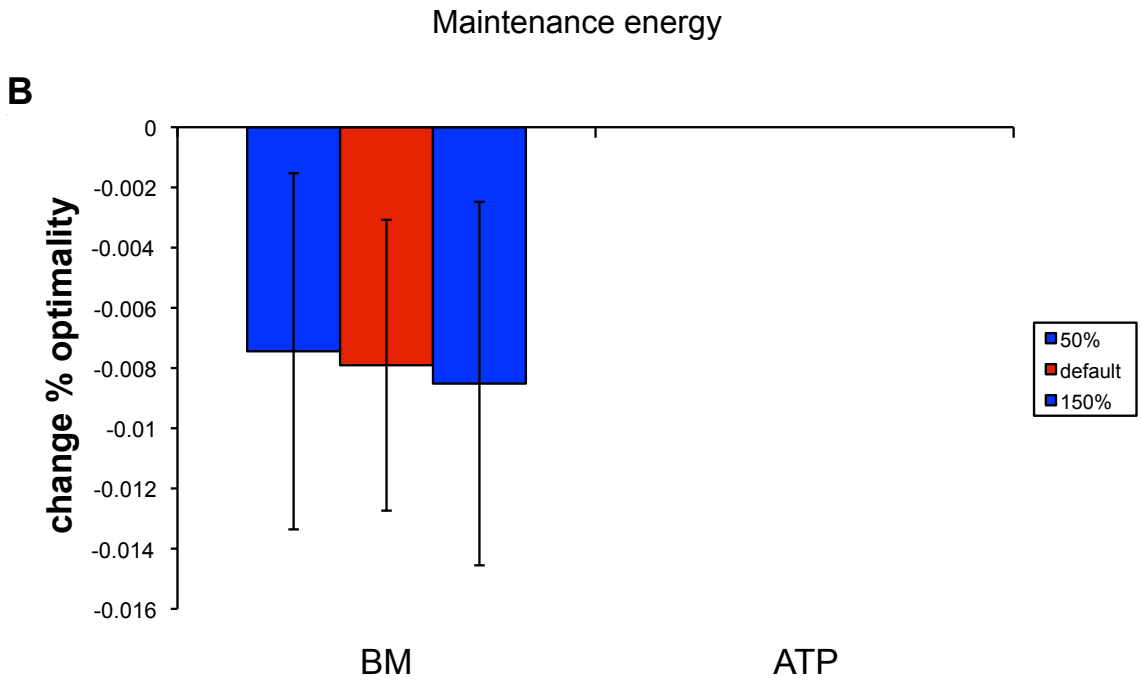

Supplement: Figure S7 — The effect that potential evolution of constraints would have on average change in % optimality between ancestor and evolved lines. A) Lipid content was altered in evolved lines from 80–120% of the default values. B) Maintenance energy in evolved lines was altered from 50–150% of the default value of 8.39 mmol/g hr. Analyses for ATP/S are not shown, as setting a lower bound on maintenance energy has no effect if ATP production is being maximized. Results for simulations run with default (red) and altered (blue) constraints are shown for the LTEE set when optimized for either BM/S or ATP/S. Error bars represent standard errors between replicate lines. (PDF) [file pcbi.1003091.s007.pdf]

Figure S8

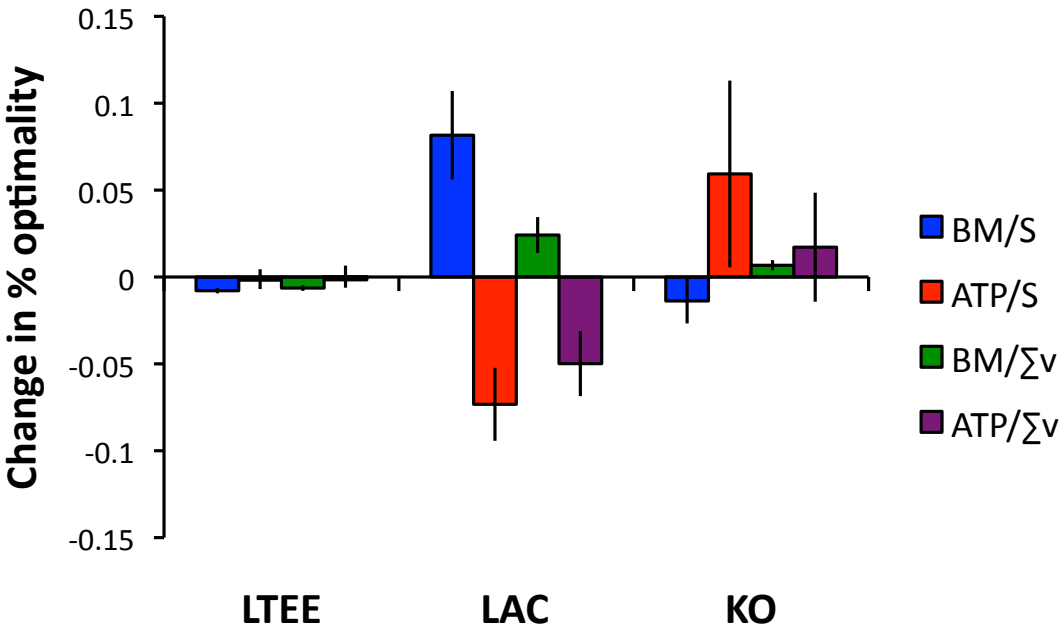

Supplement: Figure S8 — Average difference in % optimality between ancestor and evolved lines for each data set for each criterion. The criteria tested were BM/S (blue), ATP/S (red), BM/Σv (green) and ATP/Σv (purple). Error bars represent standard deviations of replicate lines. (PDF) [file pcbi.1003091.s008.pdf]
