# Supplementary material for: The Ability of Flux Balance Analysis to Predict Evolution of Central Metabolism Scales with the Initial Distance to the Optimum
Source: PLoS Comput Biol. 2013 Jun 20;9(6):e1003091. doi: 10.1371/journal.pcbi.1003091 (PMC3688462; doi:10.1371/journal.pcbi.1003091)
Supplement: Table S1 — Growth parameters for ancestral and evolved LTEE isolates. (PDF) [file pcbi.1003091.s009.pdf]

Table S1

		Glucose uptake g/g CDW h^{-1}	Acetate excretion g/g CDW h^{-1}	Growth rate h^{-1}	CDW g/g glucose
Anc	REL606	12.83 ± 0.23	3.27 ± 0.75	0.80 ± 0.10	0.36 ± 0.02
A+1	REL11392	13.59 ± 0.87	6.69 ± 0.30	1.09 ± 0.04	0.44 ± 0.02
A+2	REL11342	15.74 ± 2.24	6.69 ± 0.96	1.18 ± 0.03	0.47 ± 0.03
A+3	REL11345	16.43 ± 0.90	4.32 ± 2.86	1.22 ± 0.02	0.46 ± 0.03
A+4	REL11348	15.78 ± 2.49	6.71 ± 0.94	1.18 ± 0.05	0.45 ± 0.03
A+5	REL11367	14.80 ± 2.04	5.64 ± 1.16	1.16 ± 0.07	0.38 ± 0.02
A-1	REL11330	14.49 ± 2.13	3.67 ± 0.65	1.10 ± 0.01	0.43 ± 0.01
A-2	REL11333	16.21 ± 0.22	2.85 ± 2.07	1.29 ± 0.07	0.39 ± 0.02
A-4	REL11336	15.35 ± 1.03	2.92 ± 0.73	0.99 ± 0.06	0.41 ± 0.01
A-5	REL11339	14.22 ± 0.46	6.35 ± 0.87	1.15 ± 0.07	0.43 ± 0.01
A-6	REL11389	14.25 ± 0.67	3.16 ± 1.15	1.19 ± 0.06	0.44 ± 0.01
