# Supplementary material for: The Ability of Flux Balance Analysis to Predict Evolution of Central Metabolism Scales with the Initial Distance to the Optimum
Source: PLoS Comput Biol. 2013 Jun 20;9(6):e1003091. doi: 10.1371/journal.pcbi.1003091 (PMC3688462; doi:10.1371/journal.pcbi.1003091)
Supplement: Table S2 — Experimentally determined flux ratios for ancestral and evolved LTEE isolates. PEP through PPP is an upper bound (ub); PYR from MAL is a lower bound (lb). (PDF) [file pcbi.1003091.s010.pdf]

Table S2

	Serine through glycolysis	PYR through ED pathway	PEP through PPP (ub)	OAA from PEP	PEP from OAA	PYR from MAL (lb)
Anc	0.74 ± 0.01	0.01 ± 0.00	0.27 ± 0.05	0.69 ± 0.05	0.03 ± 0.01	0.01 ± 0.01
A+1	0.67 ± 0.01	0.06 ± 0.01	0.28 ± 0.03	0.69 ± 0.02	0.03 ± 0.01	0.02 ± 0.01
A+2	0.75 ± 0.01	0.05 ± 0.01	0.18 ± 0.03	0.61 ± 0.03	0.04 ± 0.01	0.00 ± 0.01
A+3	0.75 ± 0.01	0.13 ± 0.00	0.14 ± 0.06	0.71 ± 0.05	0.04 ± 0.02	0.03 ± 0.02
A+4	0.73 ± 0.01	0.03 ± 0.00	0.19 ± 0.05	0.59 ± 0.07	0.05 ± 0.01	0.01 ± 0.02
A+5	0.74 ± 0.01	0.03 ± 0.01	0.23 ± 0.02	0.57 ± 0.03	0.03 ± 0.02	0.02 ± 0.02
A-1	0.83 ± 0.01	0.02 ± 0.02	0.14 ± 0.04	0.59 ± 0.02	0.03 ± 0.00	0.02 ± 0.00
A-2	0.70 ± 0.02	0.03 ± 0.02	0.24 ± 0.05	0.80 ± 0.04	0.04 ± 0.01	0.00 ± 0.00
A-4	0.76 ± 0.01	0.05 ± 0.02	0.18 ± 0.03	0.63 ± 0.01	0.03 ± 0.01	0.01 ± 0.01
A-5	0.73 ± 0.01	0.03 ± 0.00	0.22 ± 0.04	0.60 ± 0.01	0.03 ± 0.02	0.02 ± 0.02
A-6	0.72 ± 0.00	0.03 ± 0.01	0.17 ± 0.05	0.58 ± 0.03	0.03 ± 0.01	0.01 ± 0.01
