# Supplementary material for: The Ability of Flux Balance Analysis to Predict Evolution of Central Metabolism Scales with the Initial Distance to the Optimum
Source: PLoS Comput Biol. 2013 Jun 20;9(6):e1003091. doi: 10.1371/journal.pcbi.1003091 (PMC3688462; doi:10.1371/journal.pcbi.1003091)
Supplement: Text S1 — Equations used to calculate flux ratios. The notation v(x) represents the flux through reaction x of the iaf1260 genome-scale model of metabolism. (PDF) [file pcbi.1003091.s011.pdf]

Text S1

Equations used to calculate flux ratios for the LTEE. The notation v(x) represents the flux through reaction x of the iaf1260 genome-scale model of metabolism.

serine through glycolysis
=2*(v(1006)+v(1043)-v(2246)-v(2203))/(v(688)+2*(v(1006)+v(1043))+v(2245)+v(2246));

pyruvate through Entner-Doudoroff
=v(688)/(v(688)+v(575)+v(1233)+v(1626)+v(1627));

oxaloacetate from phospho*enol*pyruvate
=v(2020)/(v(2020)+v(1622)+v(1623)+v(1624));

phospho*enol*pyruvate from oxaloacetate
=v(2022)/(v(2022)+v(695));

pyruvate from malate
PYR=(v(1626)+v(1627))/((v(1626)+v(1627)+v(575)+v(1233)+v(1626)+v(1627)));
